# A Case of Giant Squamous Cell Carcinoma of the Buttock Possibly Arose from Syringocystadenoma and Invaded to the Rectum

**DOI:** 10.1155/2011/213406

**Published:** 2010-10-18

**Authors:** Megumi Nishioka, Atsushi Tanemura, Takashi Yamanaka, Noriko Umegaki, Mamori Tani, Ichiro Katayama, Ichiro Takemasa, Mitsugu Sekimoto, Koichi Tomita, Noriyuki Tamai

**Affiliations:** ^1^Department of Dermatology, Course of Integrated Medicine, Graduate School of Medicine, Osaka University, 2-2 Yamadaoka, Suita-shi, Osaka 565-0871, Japan; ^2^Department of Gastroenterological Surgery, Osaka University Graduate School of Medicine, Osaka 565-0871, Japan; ^3^Department of Plastic Surgery, Osaka University Graduate School of Medicine, Osaka 565-0871, Japan; ^4^Department of Orthopaedic Surgery, Osaka University Graduate School of Medicine, Osaka 565-0871, Japan

## Abstract

We report a rare case of giant squamous cell carcinoma of the buttock infiltrated to the rectum. The tumor may have arisen from syringocystadenoma papilliferum. Since there was no sign of metastasis, radical operation including rectal amputation was performed after successful neoadjuvant therapies. Afterwards, the patient has been alive free from disease for 15 months with no lymph node and distant organ metastasis.

## 1. Introduction

Squamous cell carcinoma (SCC) of the skin is one of the most common skin cancers and likely to occur on sun exposure regions. It is prevalent in men and increases with age. The incidence of SCC is increasing these days because of longevity and increased UV exposure associated with changes of lifestyle and destruction of the ozone layer. The larger and the deeper it grows, SCC is more likely to become metastatic [1]. 

Syringocystadenoma papilliferum (SP) is an uncommon benign lesion most frequently located on the head and neck. In a series of 100 cases of SP, one case occurred on the buttock [2]. SP is categorized as a kind of epidermal nevi which are organoid nevi arising from the pluripotential germinative cells in the basal layer of embryonic epidermis. These cells give rise not only to keratinocytes but also to skin appendages. These nevi have been often classified according to their predominant component, resulting in the terms nevus verrucosus (keratinocytes), nevus sebaceous (sebaceous glands), nevus comedonicus (hair follicles), and nevus suringocytadenosus papilliferum (or SP) (apocrine glands) [3]. Occasionally, malignant tumors develop on a preexisting SP. Many of them are basal cell carcinoma [3] less frequently SCC [4] and verrucous carcinoma [5, 6].

 SCC rarely spreads from the buttock skin to the rectum although there is a report of a SCC arose from chronic perianal pyoderma that invaded around the rectum and prostate [7]. 

Here, we report a case of massive SCC of the buttock which may have originated from syringocystadenoma papilliferum and infiltrated deeply to the rectum. 

## 2. Case Report

**Figure 1:**
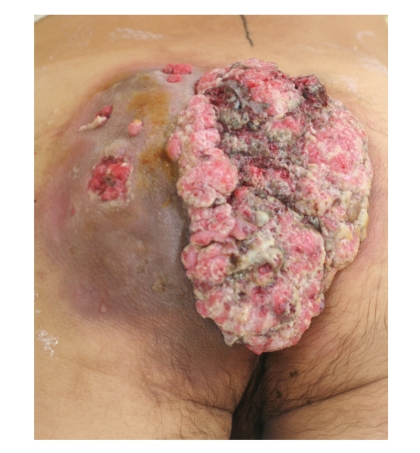
The mass on the left buttock was 20 × 10 × 4 cm in diameter and its surface was cauliflower-shaped with profuse exudates and ulceration. The skin around the mass colored brownish to purplish and had ulcers likely penetrating to the mass.

 A 48-year-old male admitted to our hospital suffering from a bulky mass on his left buttock and a foul odor. The mass on his left buttock was 20 × 10 × 4 cm in diameter, and its surface was cauliflower-shaped with profuse exudates and ulceration (Figure 1). The skin around the cauliflower-shaped mass colored brownish to purplish and partly had ulcers likely penetrating to the mass. The patient noticed a part of an elongated oval nodule in childhood and its rapid growth in his late thirties. He stated that the nodule had grown to huge.

**Figure 2:**
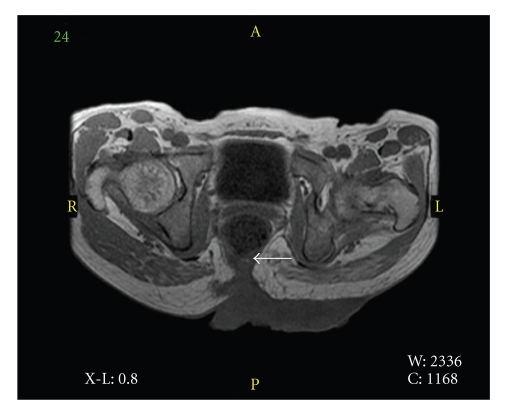
The mass invaded into coccygeal bone and the rear side of rectum (arrow).

 Computed tomography (CT) and magnetic resonance imaging (MRI) (Figure 2) showed that the mass invaded into coccygeal bone and the posterior wall of the rectum. While bilateral inguinal lymph nodes were slightly swollen, no apparent visceral metastasis was detected. 

**Figure 3:**
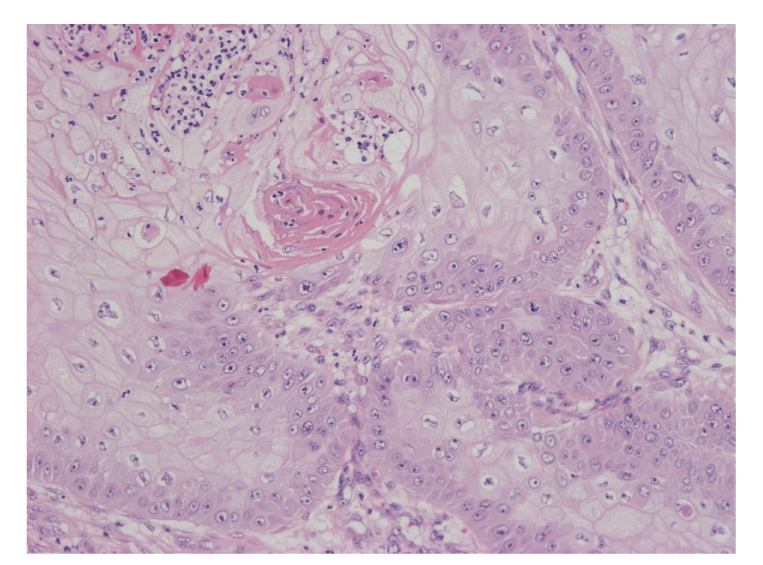
Histologically, the tumor includes papilliform-acanthosis, a number of cancer pearls, and individual cell keratinization.

 Histologically, under low magnification, the tumor cells grew upward with keratinization and downward intermingling hypertrophic scar. Under high magnification, the tumor included papilliform-acanthosis, a number of cancer pearls and individual cell keratinization (Figure 3). Atypical epidermis contained numerous mitoses and moderate to severe cell atypia. Two of bilateral inguinal lymph nodes were also biopsied as sentinel lymph nodes, and no histopathological metastasis was detected. These findings led the diagnosis of well-differentiated squamous cell carcinoma (SCC) T4N0M0 according to AJCC staging.

**Figure 4:**
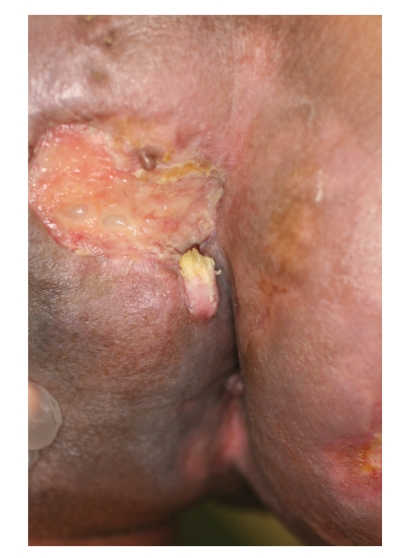
A washy red smooth nodule was noticed from the beneath of tumor after chemoradiation therapy. It was adjneent to the ulceration.

 We selected initial treatments with combination of cisplatin, fluorouracil (5-FU), and pepleomycin and concurrent 50 gray of irradiation because the tumor margin was not as demarcated as an ensured excision. As a consequence of the treatments, the tumor became necrotic and crumbled, and finally, it markedly shrank to a 7 × 3.5 cm ulcer. After chemoradiation therapy, a washy red smooth nodule was noticed from the beneath of tumor. It was adjneent to the ulceration (Figure 4). The ulcer was considered to be located at the site where the SCC originated from. The patient stated that it was the nodule that he noticed in childhood and a part of which became huge.

**Figure 5:**
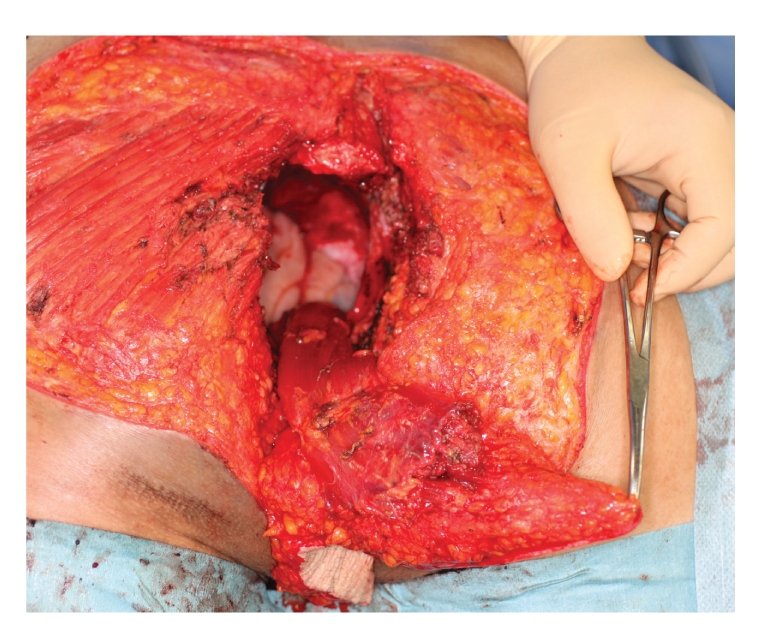
According to the first extent of tumor, buttock skin was excised together with sacrococcyx, and rectum. Reconstructive surgery by musclocutaneous flap of abdominal rectus muscle and colostomy was performed.

**Figure 6:**
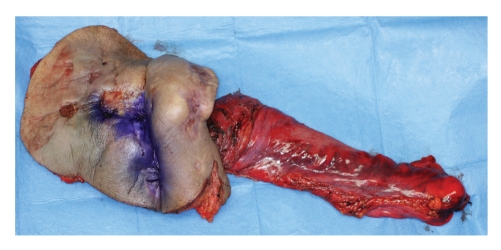
The excised specimen. The buttock skin, sacrococcyx and rectum were excised together.

 Since there is no evidence of metastasis during the above treatments, we decided to perform a radical operative procedure to achieve complete control of the tumor. According to the first extent of tumor, it was excised together with sacrococcyx and rectum. The perineum was reconstructed with a pedicled musclocutaneous flap using the right abdominal rectus muscle, and sigmoid colostoma was created (Figures 5 and 6).

**Figure 7:**
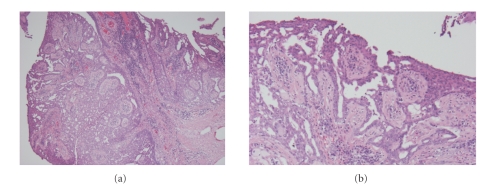
The nodule appeared after chemoradiation therapy showed papilliform structure with invaginate to the dermis and the wall was consisted with two layers of epithelial cells. The outer layer of the epithelium was composed of cuboidal cells and the inner layer of columnar cells. Many plasma cells infiltrated to the interstitial area.

 Macroscopically, the division surface of tumor showed a white fibrous tissue. Surprisingly, histopathological findings showed no remaining tissue of SCC but showed only fibrous. The fibrosis reached to rectum, and tissues was also seen around the trabecula of the sacrococcyx, suggesting previous invasion of the tumor. The nodule located behind the huge SCC showed papilliform structure which invaginated to the dermis and the wall was consisted with two layers of epithelial cells. The outer layer of the epithelium was composed of cuboidal cells and the inner layer of columnar cells. Many plasma cells infiltrated to the interstitial area (Figures 7(a) and 7(b)). According to these findings the nodule was diagnosed as SP.

 Three courses of chemotherapy with the same regimen as a neoadjuvant one were performed after surgery. Fifteen months after the operation, the patient is well and working without any local recurrence and metastasis.

## 3. Discussion

In the present case, we paid attention to the following two points. One is tumor origin, especially the relationship between long-existed SP and SCC. The other is tumor's intensive expansion and effectiveness of our treatments. 

Because buttock is not sun exposed site and our patient was relatively young among the patients who have SCC, to elucidate the origin of SCC in the present case is important. The patient and his family argued that upper part of a nodule in his buttock, which he noticed from his childhood, grew larger and became the large tumor. Indeed, the nodule appeared from the beneath of tumor after chemoradiation therapy. It was histologically diagnosed as SP. At first, we suspected that the origin of his SCC was not SP but chronic pyoderma because of the purplish pigmentation of his buttock and deep invasion [7]. However, he denied the symptom of pyoderma before development of SCC. Moreover, the purplish color was observed only in the skin lying directly on the tumor and right buttock skin was normal. Therefore, we believe that chronic pyoderma seems not to precede SCC. Cases of SP existing as a single tumor together with nevus sebaceous have been reported. And SP and nevus sebaceous are considered to share the same origin from the primary epithelial-germ. Apocrine differentiation of the germ cells leads SP, though not as well-differentiated as in sebaceous nevus [8]. And sebaceous nevus is known as an origin of many kinds of benign and malignant neoplasms including SCC [9]. Thus, the SCC in the present case may arise from sebaceous nevus existed together with SP. However, in our patient, no histopathologic features of sebaceous nevus were found. There are a few reports of SCC [4] and verrucous carcinoma [5, 6] arising from SP. Cases of syringocystadenocarcinoma papilliferum which showed histological findings of SCC are also reported [10, 11]. As a result, it is conceivable that SCC in the present case originated from SP itself, germ cells of which can differentiate to keratinocytes, although we could not determine the continuity of the SP and SCC histologically, because SCC had disappeared at the time of operation.

As for the second point, the tumor extended to the depth of rectum. To our knowledge, there was no report of SCC case treated by such a very extensive resection together with rectum and coccyx. Unexpectedly, despite this massive local invasion, no metastasis and local recurrence have been observed for long. It may suggest that chemoradiation therapy and extensive operation we performed was effective treatment for such a huge SCC. No microscopic existence of tumor cells in lymph and blood vessels may lead to a favorable clinical course.

In conclusion, we report a rare case of massive SCC of the buttock infiltrating deeply to the rectum, which was treated by extended radical operation and obtained long-term survival without local recurrence or metastasis. It was suspected that the tumor developed from SP which had existed from childhood.
